# Cuproptosis-related lncRNA signature as a prognostic tool and therapeutic target in diffuse large B cell lymphoma

**DOI:** 10.1038/s41598-024-63433-w

**Published:** 2024-06-05

**Authors:** Xiaoran Bai, Fei Lu, Shuying Li, Zhe Zhao, Nana Wang, Yanan Zhao, Guangxin Ma, Fan Zhang, Xiuhua Su, Dongmei Wang, Jingjing Ye, Peng Li, Chunyan Ji

**Affiliations:** 1 Department of Hematology, Qilu Hospital of Shandong University, Jinan, 250012 Shandong China; 2 Department of Lymphoma and Plasmacytoma Disease, Senior Department of Hematology, The Fifth Medical Center of PLA General Hospital, Beijing, China; 3 Hematology and Oncology Unit, Department of Geriatrics, Qilu Hospital of Shandong University, Jinan, 250012 Shandong China; 4 Gastroenterology Intensive Care Unit, Department of Gastroenterology, Qilu Hospital of Shandong University, Jinan, 250012 Shandong China

**Keywords:** DLBCL, Cuproptosis, lncRNA, Risk model, Immune microenvironment, Prognosis, Lymphoma, Cancer metabolism, Cancer microenvironment, Cancer models, Haematological cancer, Tumour biomarkers, Cancer

## Abstract

Cuproptosis is a newly defined form of programmed cell death that relies on mitochondria respiration. Long noncoding RNAs (lncRNAs) play crucial roles in tumorigenesis and metastasis. However, whether cuproptosis-related lncRNAs are involved in the pathogenesis of diffuse large B cell lymphoma (DLBCL) remains unclear. This study aimed to identify the prognostic signatures of cuproptosis-related lncRNAs in DLBCL and investigate their potential molecular functions. RNA-Seq data and clinical information for DLBCL were collected from The Cancer Genome Atlas (TCGA) and Gene Expression Omnibus (GEO). Cuproptosis-related lncRNAs were screened out through Pearson correlation analysis. Utilizing univariate Cox, least absolute shrinkage and selection operator (Lasso) and multivariate Cox regression analysis, we identified seven cuproptosis-related lncRNAs and developed a risk prediction model to evaluate its prognostic value across multiple groups. GO and KEGG functional analyses, single-sample GSEA (ssGSEA), and the ESTIMATE algorithm were used to analyze the mechanisms and immune status between the different risk groups. Additionally, drug sensitivity analysis identified drugs with potential efficacy in DLBCL. Finally, the protein–protein interaction (PPI) network were constructed based on the weighted gene co-expression network analysis (WGCNA). We identified a set of seven cuproptosis-related lncRNAs including LINC00294, RNF139-AS1, LINC00654, WWC2-AS2, LINC00661, LINC01165 and LINC01398, based on which we constructed a risk model for DLBCL. The high-risk group was associated with shorter survival time than the low-risk group, and the signature-based risk score demonstrated superior prognostic ability for DLBCL patients compared to traditional clinical features. By analyzing the immune landscapes between two groups, we found that immunosuppressive cell types were significantly increased in high-risk DLBCL group. Moreover, functional enrichment analysis highlighted the association of differentially expressed genes with metabolic, inflammatory and immune-related pathways in DLBCL patients. We also found that the high-risk group showed more sensitivity to vinorelbine and pyrimethamine. A cuproptosis-related lncRNA signature was established to predict the prognosis and provide insights into potential therapeutic strategies for DLBCL patients.

## Introduction

Diffuse large B cell lymphoma (DLBCL) is the most common type of non-Hodgkin’s lymphoma. Due to the high heterogeneity of DLBCL, treatment response and prognosis vary widely among DLBCL patients^1^. Currently, the first-line treatment regimen for DLBCL patients is R-CHOP, including rituximab plus cyclophosphamide, doxorubicin, vincristine and prednisone^2^. Approximately 60% of patients with DLBCL achieve remission following R-CHOP regimen, while around 40% of patients remain unresponsive and 10–15% are classified as primary refractory cases^3^. At present, the International Prognostic Index (IPI) is the predominant method utilized to assess prognosis and guide treatment decisions for patients with DLBCL. However, IPI relies only on clinical factors and may not provide a comprehensive forecast of patient outcomes. In recent years, with the advancements in high-throughput sequencing technology, an increasing number of novel indicators with potential predictive value for DLBCL progression have been identified. Studies suggest that incorporating molecular features and tumor microenvironment (TME) into the IPI may be helpful to characterize the signatures of recurrent and refractory lymphoma and select more appropriate treatment^4^. Therefore, there is an urgent need to develop more comprehensive risk models for DLBCL patient.

Copper is a vital nutrient, however, an excess of copper can lead to oxidative stress and cytotoxicity, which is found to be closely related to tumor initiation, development and metastasis^5–7^. Higher levels of copper has been found in malignant tumors compared with normal tissues, indicating its potential role in tumor advancement^8^. Similar results were found in lymphoma. Higher levels of copper was found in serum and lymphoma tissue compared with normal tissue ^9^. Besides, serum copper levels have been identified as an independent prognostic factor for lymphoma patients, and are closely related to tumor activity^10,11^. Copper ion carrier and copper chelator exhibit cancer-resistant properties. Studies have shown that copper compounds can induce apoptosis in lymphoma cell lines in vitro and reduce the growth of lymphoma in vivo^12–14^. The copper chelating agent ATN-224 has demonstrated the ability to induce caspase-independent cell death in DLBCL, which is expected to be a new treatment for relapsed and refractory DLBCL^15^. Cuproptosis, a new type of programmed cell death, differs from other cell death pathways such as apoptosis, ferroptosis, and necroptosis^16^. It is demonstrated that cuproptosis is initiated by the direct interaction of copper with lipoylated components of the tricarboxylic acid (TCA) cycle, which leads to the aggregation of lipid acylated proteins and subsequent depletion of iron-sulfur cluster proteins. This cascade of events results in proteotoxic stress and cell death ultimately^16^. Cuproptosis is believed to play a crucial role in tumor suppression, highlighting its potential for targeted anti-cancer therapy. A recent study has shown that cupreous nanomaterials can induce cuproptosis in bladder cancer^17^. Combining nanomedicine that can induce cuproptosis with Programmed Cell Death-Ligand 1 (PD-L1) antibody can enhance bladder cancer therapy^18^. These findings provide a novel strategy for future cancer therapy. Furthermore, the expression of cuproptosis related genes in lymphoma has also been detected^19^. Therefore, further research on cuproptosis-related pathways and their regulatory molecular mechanisms is warranted and of great significance.

Long noncoding RNAs (lncRNAs), a group of non-coding RNAs longer than 200 nucleotides, play an important role in immune response, including immune cells infiltration, antigen recognition, antigen exposure and tumor suppression^20^. Extensive research has demonstrated the association between IncRNAs and tumor proliferation, invasion, metastasis and prognosis^21^. Moreover, studies have showed the role of lncRNAs in the pathogenesis and progression of DLBCL. For example, it has been demonstrated that the ALKBH5-mediated N6-methyladenosine modification of TRERNA1 promotes proliferation of DLBCL cells via p21 downregulation^22^. LINC00461 was shown to inhibit apoptosis and promoting recurrence in DLBCL through miR-411-5p/BNIP3 pathway^23^.Through meta-analysis, Xu et al. found that lncRNA expression profile could aid in the diagnosis and classification of DLBCL, and abnormal lncRNA expression level was associated with poor prognosis of DLBCL patients, indicating that lncRNAs can be used as candidate prognostic biomarkers for DLBCL^24^.

At present, the pathogenesis of DLBCL remains incompletely understood. Studies indicates the important role of lncRNA in DLBCL. Cuproptosis-related genes expression levels are associated with the prognosis of DLBCL patients, but their function has not been fully elucidated. It is important to identify cuproptosis-related lncRNAs with prognostic value in DLBCL patients. In this study, we investigated the relationship between cuproptosis-related lncRNAs and DLBCL using bioinformatics analysis. Our findings may provide novel insights into clinical individual treatment of DLBCL.

## Materials and methods

### Data collection

RNA sequence transcriptome data, clinical information of DLBCL were downloaded from TCGA database (https://portal.gdc.cancer.gov/) and GEO database (https://www.ncbi.nlm.nih.gov/geo/). The data of 47 DLBCL samples with a complete mRNA expression profile were obtained from the TCGA database. 414 samples with a complete mRNA expression profile are involved in GSE10846. The transcriptome information was collected in the fragment per kilobase million (FPKM) format that has been normalized through the Perl programming language (version Strawberry-Perl-5.30.0; https://www.perl.org). Meanwhile, DLBCL patients with missing survival information were excluded from the study.

### Identification of cuproptosis-related lncRNAs

First, we obtained ten cuproptosis-related genes from previous literature, including seven positive hits (FDX1, LIAS, LIPT1, DLD, DLAT, PDHA1 and PDHB) and three negative hits (MTF1, GLS and CDKN2A). In order to assess the association between the cuproptosis-related lncRNAs and genes, we used the “ggalluvial” package in R, and identified lncRNAs related with cuproptosis by Pearson correlation analysis (|R|> 0.3, *P* < 0.001).

### Construction and validation of cuproptosis-related risk model

We randomly divided (1:1 ratio) the 414 cases extracted from the GSE10846 dataset into a training set (n = 207) and a validation set (n = 207). A separate 47-patient TCGA data set was used as an external validation set to evaluate the predictive efficacy and robustness of the risk model. We made use of univariate Cox regression analysis to obtain prognostic cuproptosis-related lncRNAs in GSE10846 dataset. Lasso regression analysis was applied to reduce the dimension of high-latitude data using “glmnet” package of R language. Ten-fold cross-validation was employed to avoid the overfitting problem and select the penalty parameter (λ) according to the minimum criteria. Then, multivariate Cox regression analysis was conducted to identify candidate prognostic cuproptosis-related lncRNAs and we calculated the cuproptosis risk score for DLBCL patients. The risk score for each DLBCL patient was counted with the following algorithm:

$$\text{Risk score}={\sum }\text{Expi}\times \beta \text{i}$$

(βi stands for each lncRNA coefficient, and Expi stands for each lncRNA presentation). Thereafter, the DLBCL patients were divided into low- and high-risk groups according to the median risk score. The Kaplan–Meier survival curve combined with a log-rank test was used to estimate the difference in overall survival (OS) between patients in low-risk and high-risk groups using R packages “survival”. The principal component analysis (PCA) was used to investigate the distribution pattern of patients in low- and high-risk groups based on the prognostic cuproptosis-related lncRNAs using R package “ggplot2”. The pheatmap package in R was used to generate heatmap profiles of the expressed candidate prognostic lncRNAs.

### Independent prognostic factor analysis and nomogram

Univariate and multivariate Cox regression analysis were performed to verify whether the risk model was an independent prognostic indicator for DLBCL patients using R package “survival”. A nomogram model was constructed of clinicopathological characteristic and risk score via R package “RMS”, which can better predict the 1-, 3- and 5-year’s survival probability of patients. Additionally, the prognostic capability of the risk model constructed by risk score was validated using time-dependent receiver operating characteristic (ROC) analysis via R package “time ROC”. The calibration diagram was commonly tool to assess the accuracy of the nomogram. Kaplan–Meier analysis was used to evaluate survival distinction between low-risk and high-risk patients with the same clinicopathological characteristics.

### Immune infiltration landscape analysis

The CIBERSORT algorithm was utilized to investigate the immune infiltration landscape, and 22-types immune cells were evaluated based on “CIBERSORT R script v1.03”. A single sample gene set enrichment analysis (ssGSEA) algorithm was performed to assess the proportion of 23-types of immune cells via the “GSVA” R package.

### Functional enrichment analysis

The genes differentially expressed between the high-risk and low-risk groups were identified (|log2(fold change)|> 1 and FDR < 0.05) with the “edgeR” R package and functionally annotated based on the Gene Ontology (GO) and the Kyoto Encyclopedia of Genes and Genomes (KEGG) with the ‘clusterProfiler’ R package (adjusted *P-*value < 0.05).

### Drug sensitivity prediction

The “pRRophetic” R package was used to predict the Drug sensitivity (IC50) of chemotherapy drugs. The chemotherapeutic medications were obtained from the Genomics of Drug Sensitivity in Cancer (GDSC).

### Construction of protein–protein interaction network (PPI) based on weighted gene co-expression network analysis (WGCNA)

The cuproptosis-related lncRNAs and cuproptosis-related mRNA modules with the highest correlation coefficients were first screened by WGCNA and then the relationship matrix and adjacence matrix were constructed in turn ^25^.The topological overlap matrix was constructed using a power value of seven. Subsequently, the correlation between these modules and traits was analyzed based on the Pearson correlation coefficient. The genes in the MEturquoise module were included for further analysis and the edges with the top 500 weight values were selected and imported into Cytoscape for the construction of a visualized PPI network. Meanwhile, Cytoscape’s MCODE plugin was used to extract the central genes from the PPI network.

### Statistical analysis

All statistical analyses were performed using R software (version 4.2.0) and Perl software (strawberry-Perl-5.30.0). Spearman-ranked correlation analysis was applied to investigate the correlation between risk score and IC50, with *P*-value < 0.05 was considered significantly different. Differential functions were analyzed using the Wilcoxon rank-sum test between the two groups, and statistical significance was set at *P*-value < 0.05.

### Ethical approval and consent to participate

The study complied with the principles of the Declaration of Helsinki. Access to the de-identified linked dataset was obtained from the TCGA and GEO databases in accordance with the database policy. For analyses of de-identified data from the TCGA and GEO databases, institutional review board approval and informed consent were not required.

## Results

The detailed flow chart of the study is displayed in Supplementary Fig. 1

### Identification of prognostic cuproptosis-related lncRNAs

**Figure 1:**
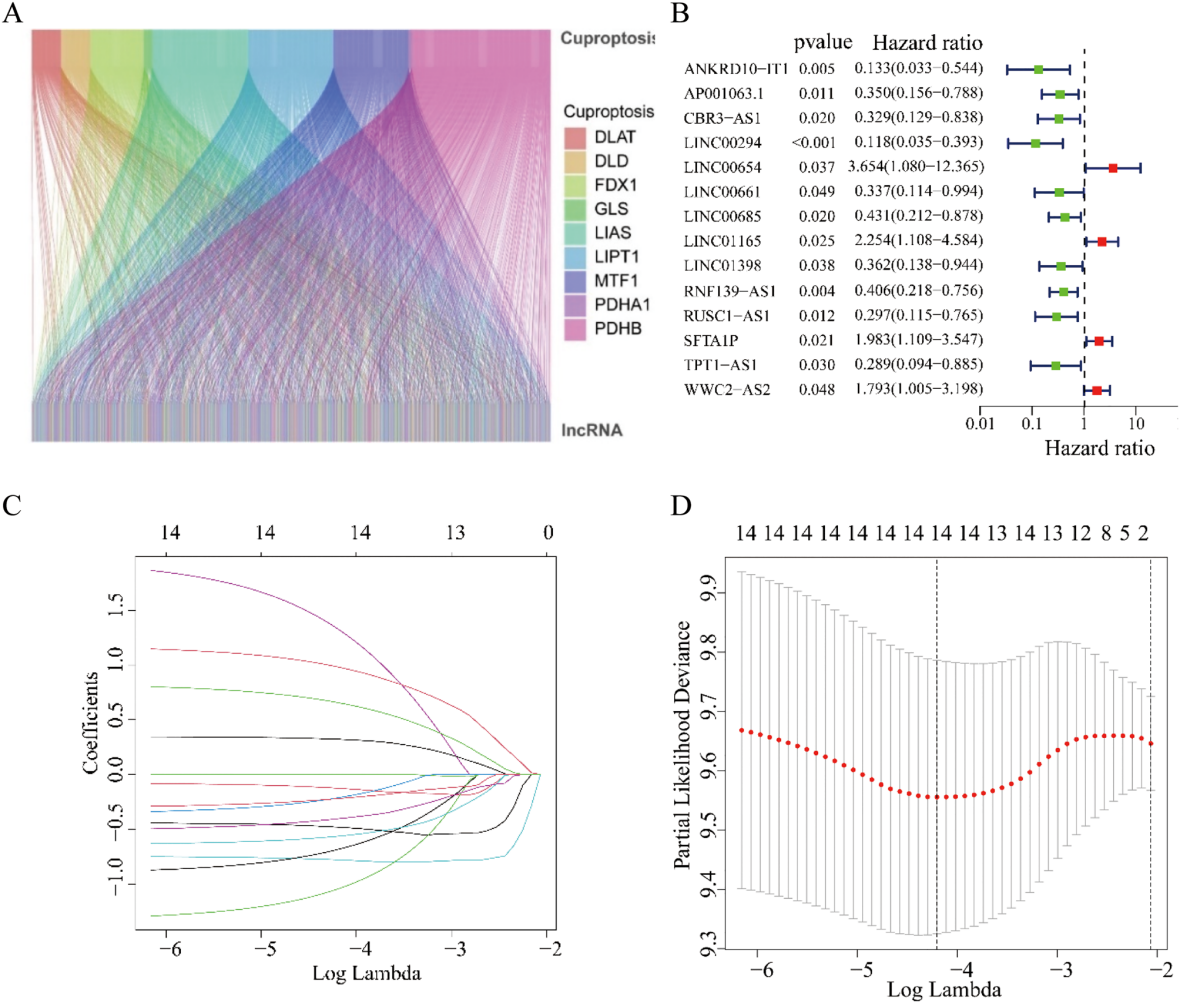
Identification of prognostic cuproptosis-related lncRNAs in DLBCL. **(A)** The co-expression analysis of 10 cuproptosis-associated genes and cuproptosis-related lncRNAs. **(B)** Univariate Cox regression analysis suggested that 14 cuproptosis-related lncRNAs are associated with OS in DLBCL. **(C,D)** Lasso regression analysis identified the minimal lambda and coefficients of prognostic cuproptosis-related lncRNAs.

We analyzed cuproptosis-related genes and obtained 381 lncRNAs associated with cuproptosis through Pearson correlation analysis (Fig. 1A). By univariate Cox regression analysis, we found 14 cuproptosis-related lncRNAs that were significantly associated with outcomes of DLBCL patients and LINC00654, LINC01165, SFTA1P, WWC2-AS2 were high risk lncRNAs (Fig. 1B). We further conducted Lasso regression analysis to narrow down the cuproptosis-related lncRNAs, and 14 cuproptosis-related lncRNAs were obtained by the λ minimization method (Fig. 1C, D).

### Risk model construction of cuproptosis-related lncRNAs

**Figure 2:**
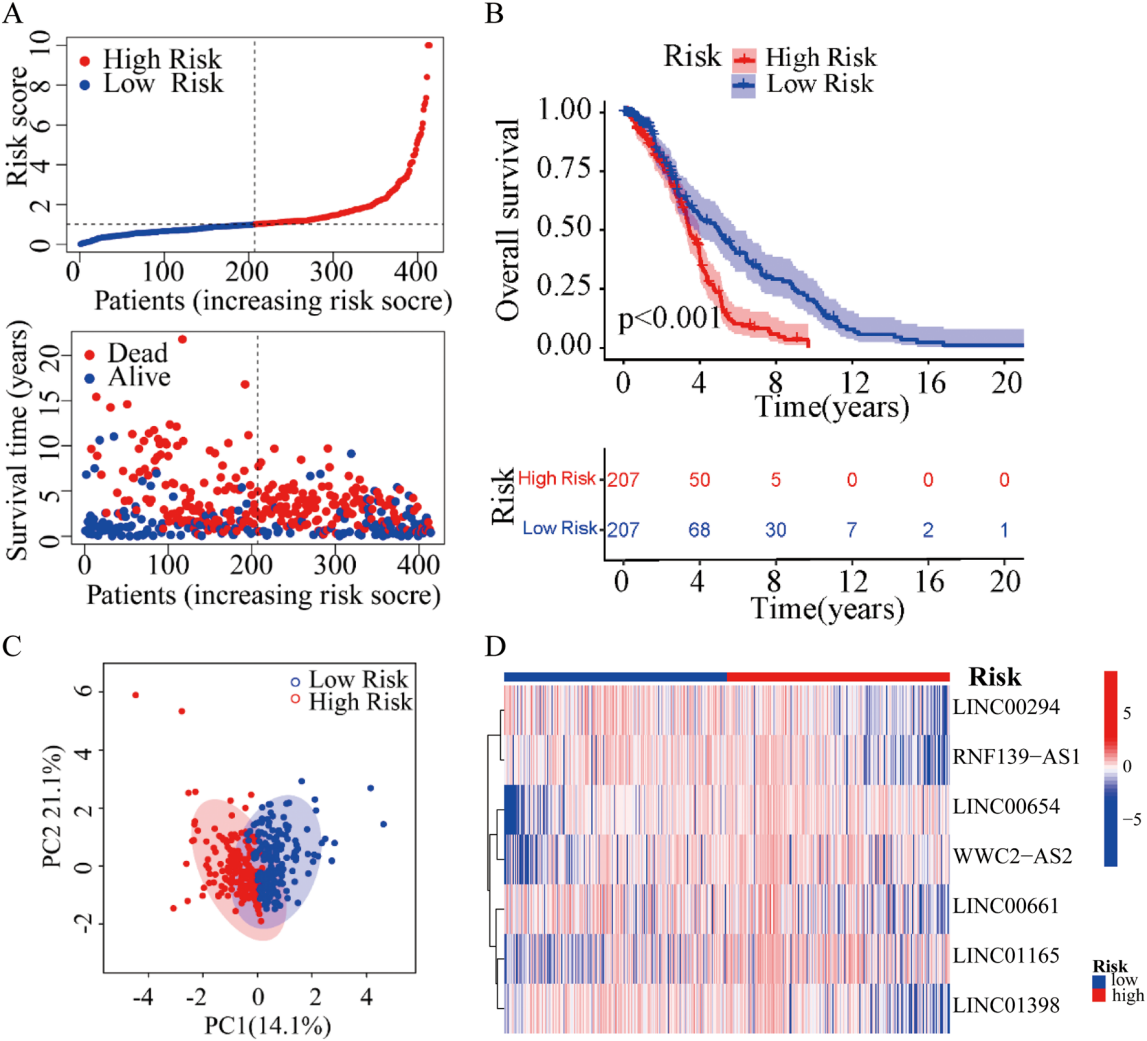
Construction of risk model based on the prognostic 7 cuproptosis-related lncRNAs of patients with DLBCL. **(A)** Distribution of risk scores and survival status and survival time patterns of each sample. **(B)** The Kaplan–Meier survival analysis of the high-risk and low-risk groups based on the risk model and median risk score. **(C)** PCA analysis between the low-risk and high-risk groups based on the risk model of the 7 cuproptosis-related lncRNAs expression profiles. **(D)** The heatmap of the expression levels of 7 cuproptosis-related lncRNAs in the high-risk and low-risk groups.

Based on the multivariate Cox regression analysis, seven cuproptosis-related lncRNAs were identified to construct the risk model. In GSE10846 set, the risk score of each DLBCL patient was calculated, and the patients were divided into the low- and high-risk group based on the median risk score. Risk score = LINC00654 × 1.79 − LINC00294 × 1.42 − LINC00661 × 0.98 + LINC01165 × 1.10 − LINC01398 × 1.47 − RNF139-AS1 × 1.26 + WWC2-AS2 × 0.75. We investigated the distribution of risk scores, survival time and survival status between high- and low-risk groups in DLBCL patients (Fig. 2A). The Kaplan–Meier survival curve suggested that the OS rate of DLBCL patients in low-risk group was significantly higher than that in high-risk group (Fig. 2B), suggesting that the risk score had prognostic value for DLBCL patients. Principal component analysis (PCA) illustrated a clear separation between low- and high-risk groups based on the seven cuproptosis-related lncRNAs (Fig. 2C). In addition, the relative expression of seven cuproptosis-related lncRNAs was calculated in DLBCL patients. Compared with the low-risk group, patients in the high-risk group have higher expression levels of three cuproptosis-related lncRNAs including LINC00654, WWC2-AS2 and LINC01165, which were high-risk lncRNAs. (Fig. 2D).

### Verification of the cuproptosis-related lncRNA risk model

**Figure 3:**
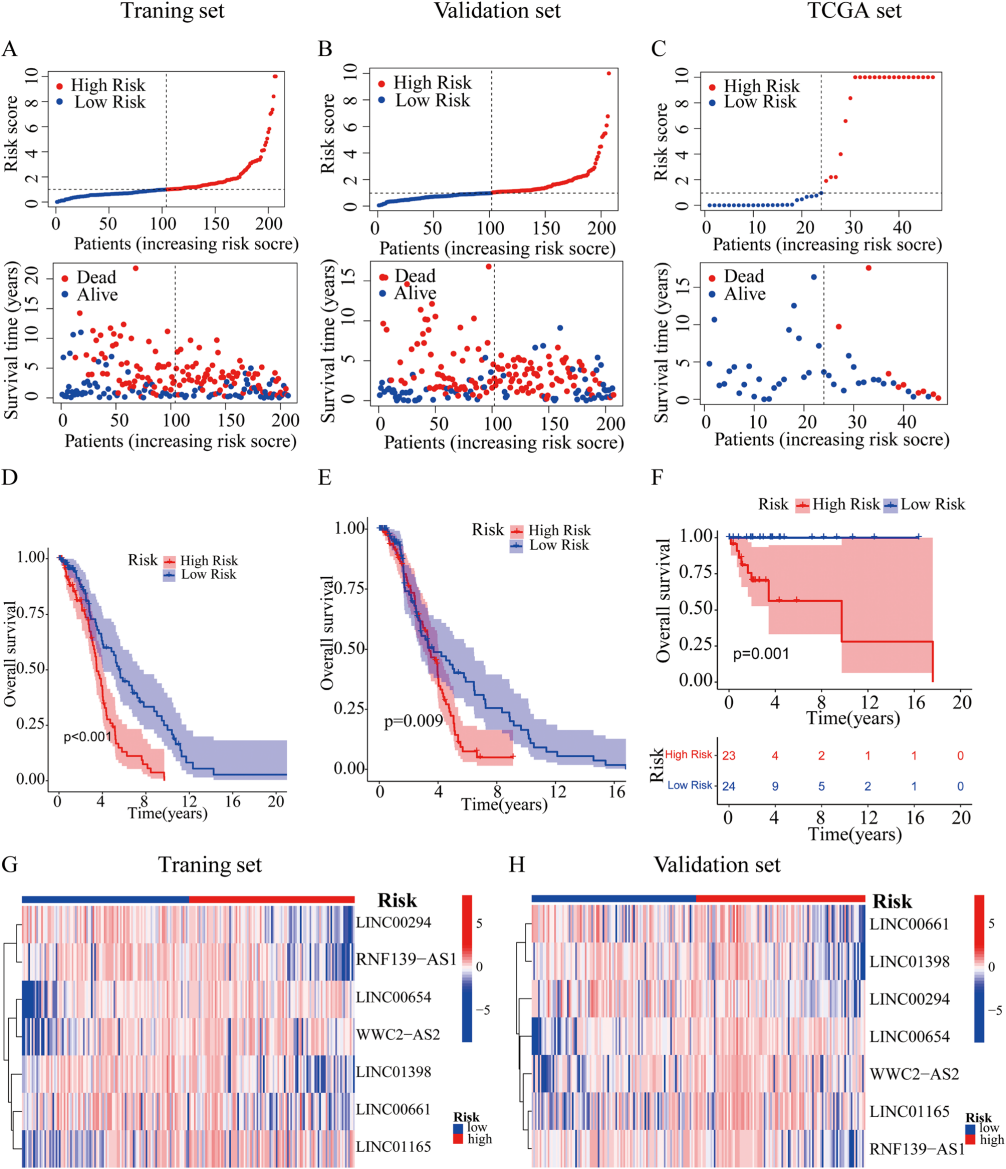
Validation of the lncRNA-based prognostic risk model in the training set and validation set and TCGA set. Risk score distribution of patients with DLBCL based on cuproptsis-related lncRNAs and scatter dot plot showed the association between the risk score distribution and the survival time in training set **(A)**, validation set **(B)** and TCGA set **(C)**. The Kaplan–Meier survival curve displayed the OS rate in DLBCL patients in training set **(D)**, validation set **(E)** and TCGA set **(F)**. Heat maps of expression of the 7 lncRNAs signals associated with cuproptosis in training set **(G)** and validation set **(H)**.

We applied the TCGA set and the GSE10846 set to test the reliability of the established risk model. The patients with DLBCL in GSE10846 set were randomly divided into training and validation set depended on the seven cuproptosis-related lncRNAs prognostic signature. Meanwhile, TCGA set was employed to further investigate the precision of risk model. Using the method mentioned before, we calculated the risk score for each patient in training, validation and TCGA set, and reclassified patients into low-risk and high-risk groups based on the median risk score. The distribution of risk scores, the pattern of survival status, the survival time, as well as the expression of the seven cuproptosis-related lncRNAs in the training set (Fig. 3A, G), the validation set (Fig. 3B, H) and the TCGA set (Fig. 3C). Compared with patients in the low-risk group, patients in the high-risk group had lower expression levels of four cuproptosis-related lncRNAs including LINC00294, RNF139-AS1, LINC01398 and LINC00661, indicating that the four cuproptosis-related lncRNAs were low-risk lncRNAs; patients in the high-risk group had higher expression levels of three cuproptosis-related lncRNAs including LINC00654, WWC2-AS2 and LINC01165, indicating that the three cuproptosis-related lncRNAs were high-risk lncRNAs (Fig. 3G, H). Not surprisingly, Kaplan–Meier survival analysis also showed that patients with high-risk score had a worse OS than those with low-risk score (*P*_traning set_ < 0.001, *P*_validation set_ = 0.009, *P*_TCGA_ = 0.001, Fig. 3D–F). The above results demonstrated that the risk model based on seven cuproptosis-related lncRNAs could accurately predict DLBCL prognosis.

### Nomogram and correlation of the risk model with clinical characteristics

**Figure 4:**
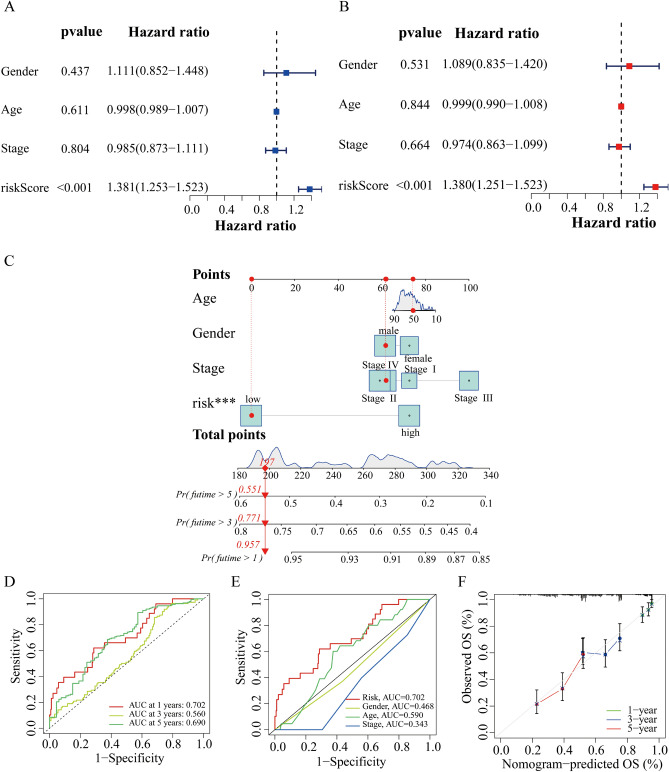
Construction and validation of the cuproptosis-related clinicopathological nomogram model. **(A)** Univariate Cox regression analysis of OS rate of DLBCL patients and clinical parameters. **(B)** Multivariate Cox regression analysis of OS rate of DLBCL patients and clinical parameters. **(C)** A prognostic nomogram for predicting the 1-, 3- and 5-year OS of DLBCL patients. **(D)** ROC curve of the predictive characteristic and AUC of 1-, 3-, and 5-year survival. **(E)** AUC of ROC curves showing the prognostic accuracy of the risk score and other prognostic factors in DLBCL patients. **(F)** The calibration curve evaluating the accuracy of the nomogram model. ***P < 0.001.

First, we used independent analysis to investigate whether the risk score based on cuproptosis-related lncRNAs could be a prognosis factor for DLBCL patients. Univariate and multivariate Cox regression analysis showed that risk scores were independent prognostic factors of the OS in DLBCL patients (Fig. 4A, B). Nomograms is widely used to predict the survival and prognosis of cancer patients, so we constructed the prognostic nomogram based on the clinical characteristics and risk score to predict individual 1-, 3-, and 5-year OS (Fig. 4C). In the time-dependent ROC curve, the 1-, 3- and 5-year survival AUCs were 0.702, 0.560, 0.690, which showed good predictive performance (Fig. 4D). Similarly, risk score accounted for the largest area of the Roc curve (AUCs = 0.702), indicating that this model has a high discriminant ability (Fig. 4E). The calibration curve was drawn in Fig. 4F which indicated the accurate coincidence of nomogram in DLBCL patients. Collectively, all these data demonstrated the accuracy and validity of nomogram.

### Correlation analysis of cuproptosis-related prognostic signature and the clinicopathological characteristics in DLBCL patients

**Figure 5:**
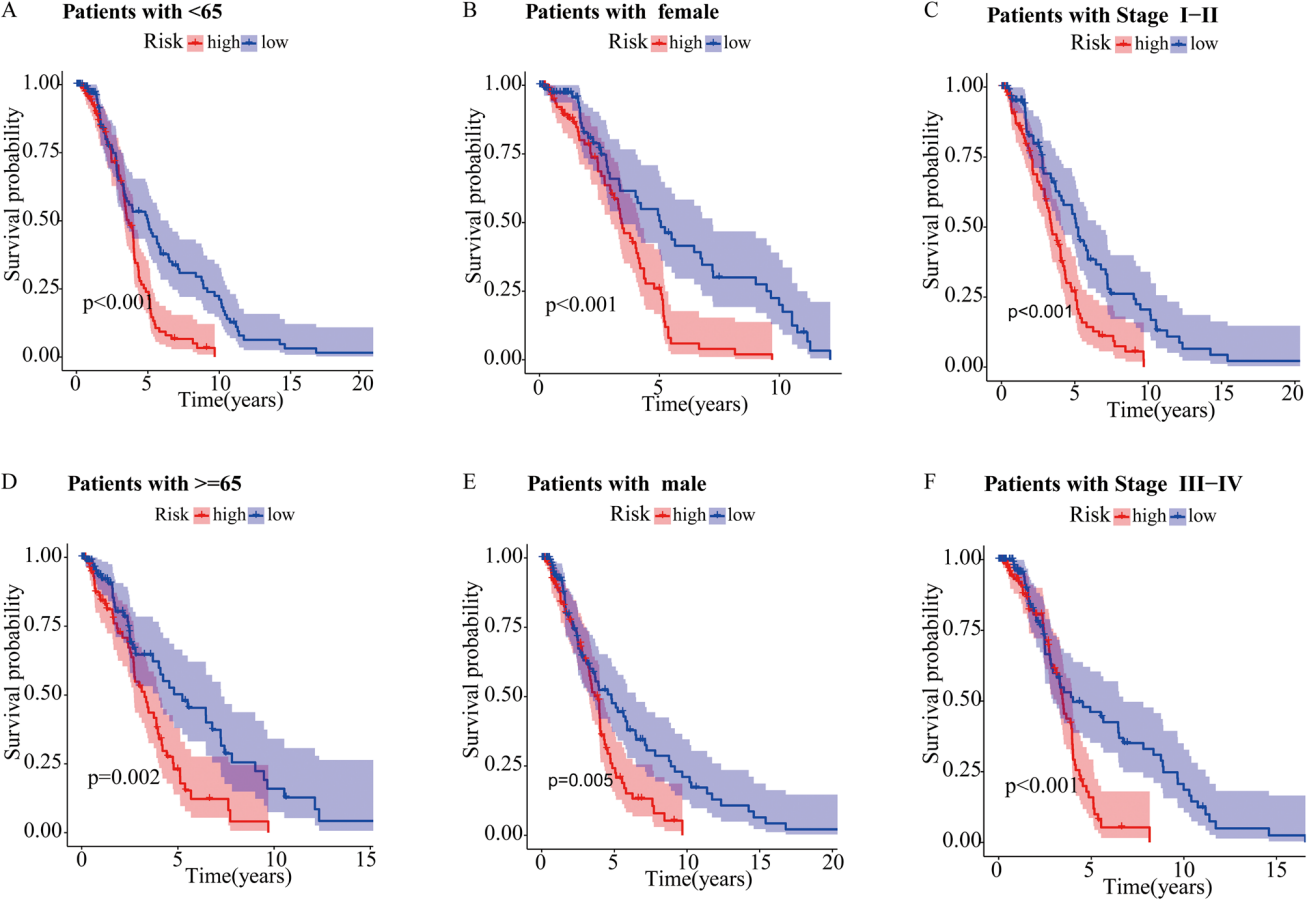
The Kaplan–Meier survival curve of high-risk and low-risk groups in the patients stratified by different clinicopathological characteristics. age **(A,B)**, gender **(C,D)**, and stage **(E,F)**.

Because of the clinicopathological heterogeneity of DLBCL, we conducted a stratified subgroup analysis to further investigate whether there was a significant relevance between the cuproptosis-related prognostic signature and clinical features. Patients with DLBCL were grouped according to the age, gender and stage. We found that the survival time of patients in the high-risk group was significantly shorter than those in the low-risk group (Fig. 5A–F). Therefore, the prognostic signature based on the cuproptosis-related lncRNA could accurately predict the prognosis of DLBCL patients, regardless of clinicopathological heterogeneity.

### Immune infiltration landscape analysis in the cuproptosis-related model

**Figure 6:**
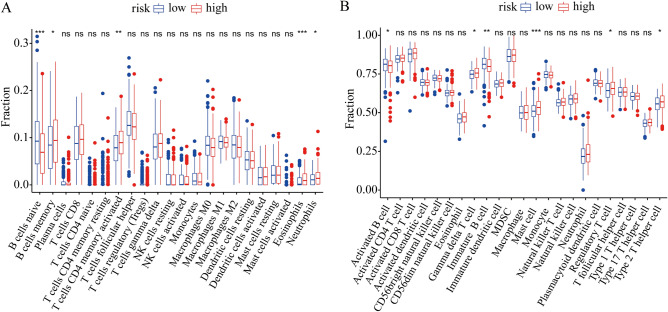
Immune infiltration landscape analysis of DLBCL patients in low- and high-risk groups. **(A)** The CIBERSORT algorithm was used to calculate the fraction of infiltration of 22 immune cells in the high-risk and low-risk groups. **(B)** The ssGSEA algorithm was used to calculate the fraction of infiltration of 23 immune cells in the high-risk and low-risk groups. *P < 0.05, **P < 0.01, and ***P < 0.001.

To investigate the effect of cuproptosis on DLBCL tumor immune environment, CIBERSORT and ssGSEA were employed to evaluate the infiltration of immune cells. The CIBERSORT algorithm showed that there was a remarkable increase of the proportion of memory B cells, activated memory CD4 T cells, eosinophils and neutrophils in the high-risk group than low-risk group, while the naïve B cells was lower in the high-risk group (Fig. 6A). The ssGSEA algorithm showed that the percentage of immature B cells and activated B cells significantly decreased in high-risk groups, while gamma delta T cells, mast cells, regulatory T cells and type 2 T helper cells were significantly higher in the high-risk group (Fig. 6B).

### Functional enrichment analysis of the differently expressed genes

**Figure 7:**
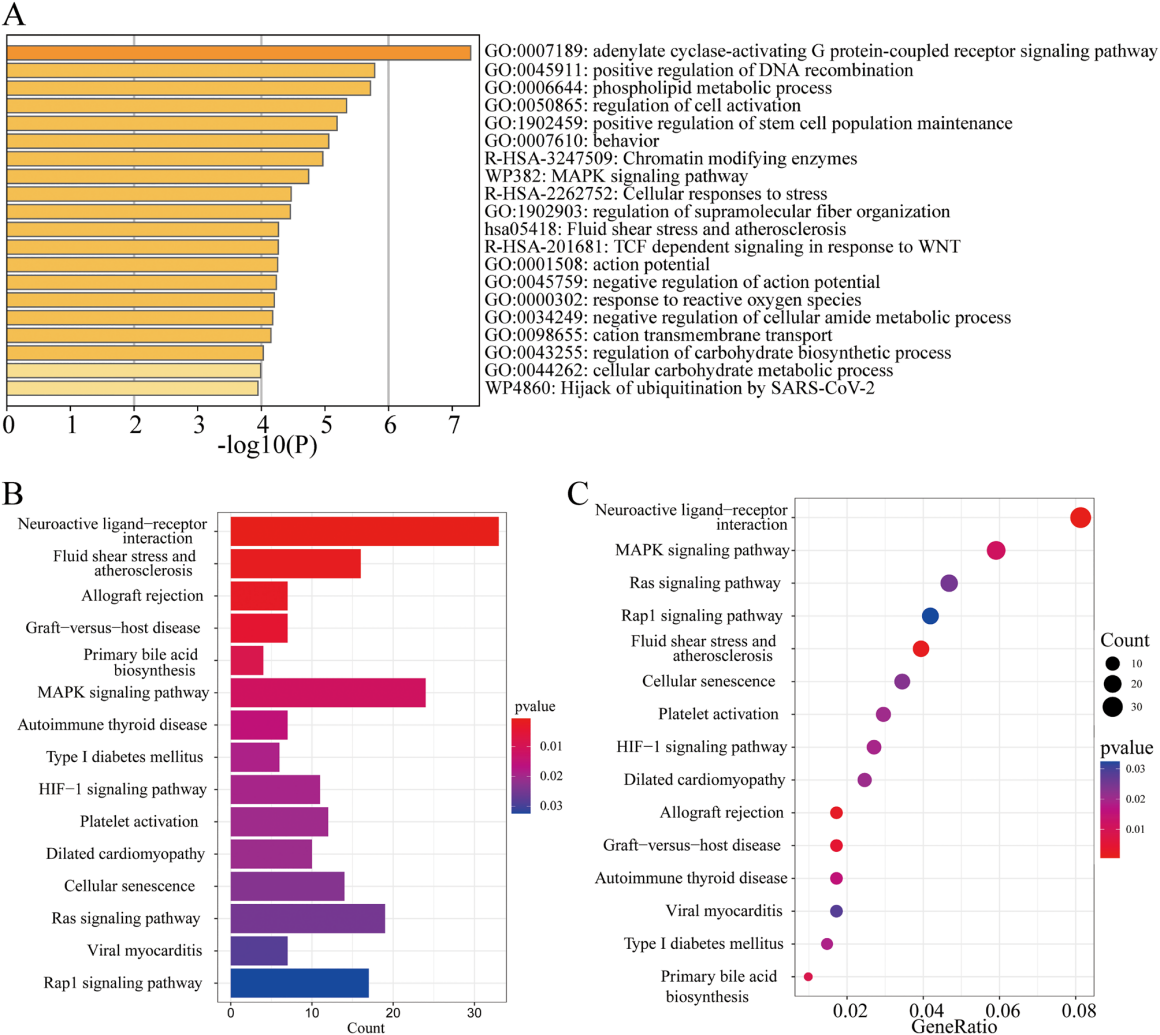
Functional enrichment analysis of the differential expressed genes. **(A)** GO analysis based on differentially expressed gene between high- and low-risk groups. **(B,C)** KEGG analysis showing the results of biological processes enrichment terms for differentially expressed genes.

GO and KEGG pathway enrichment analysis was performed to explore potential bio-functions and signaling pathways of differentially expressed genes among high-risk and low-risk groups. The GO analysis showed that differentially expressed genes were significantly enriched in adenylate cyclase-activating G protein-coupled receptor signaling pathway, positive regulation of DNA recombination, phospholipid metabolic process, regulation of cell activation, positive regulation of stem cell population maintenance, MAPK signaling pathway, cellular responses to stress, regulation of supramolecular fiber organization, fluid shear stress and atherosclerosis, response to reactive oxygen species, negative regulation of cellular amide metabolic process and cation transmembrane transport (Fig. 7A). In addition, the KEGG analysis showed that differentially expressed genes were mainly enriched in neuroactive ligand-receptor interaction, MAPK signaling pathway, fluid shear stress and atherosclerosis, allograft rejection, graft-versus-host disease, primary bile acid biosynthesis (Fig. 7B, C). Our conclusion indicated that cuproptosis may have a closely correlation with signal transduction, metabolism, inflammation and immune-related pathways in DLBCL.

### Analysis of drug sensitivity in low- and high-risk groups

**Figure 8:**
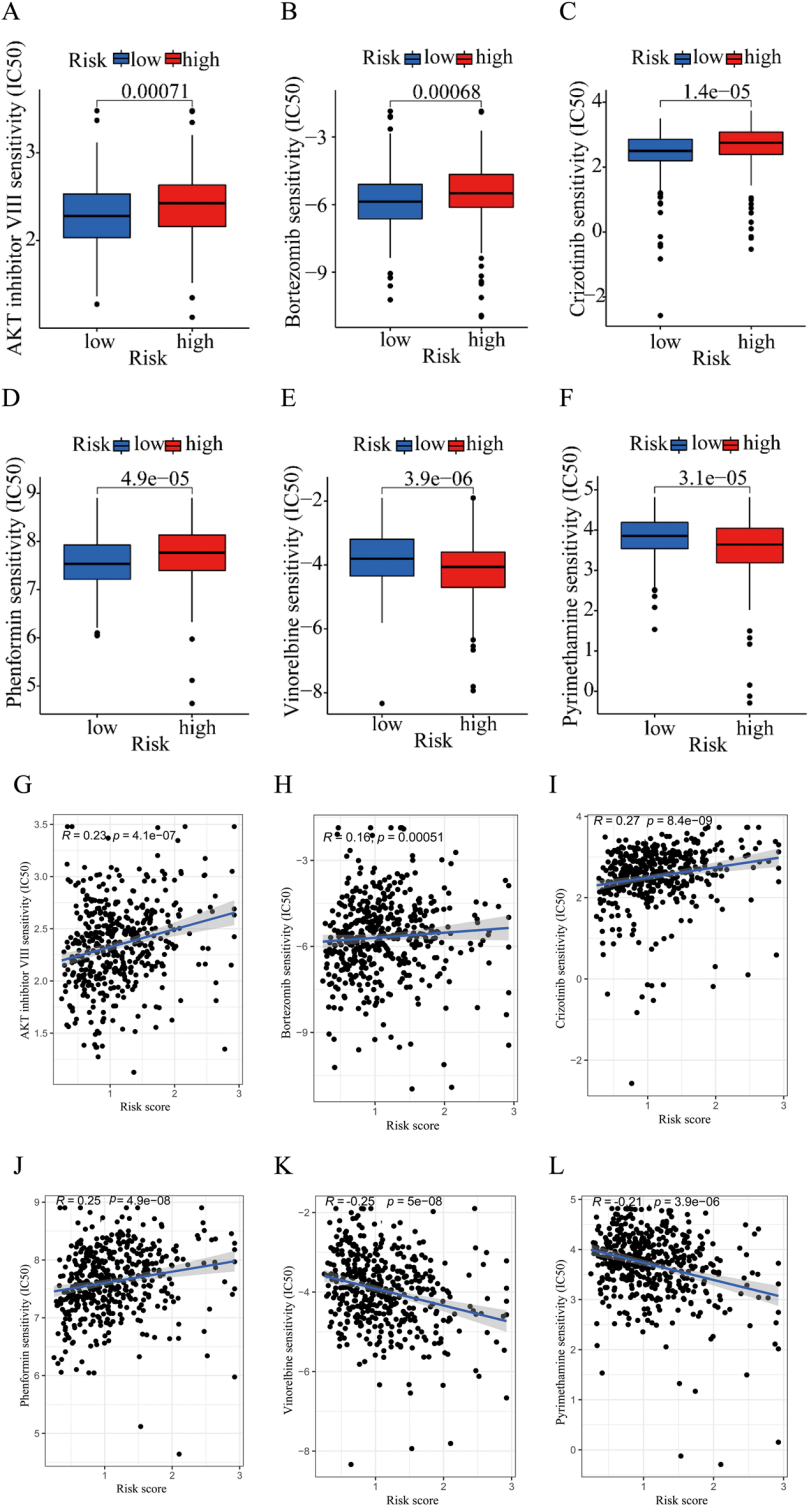
Drug sensitivity analysis in low- and high-risk group. The IC50 value exhibits a significant difference in low- and high-risk group among AKT inhibitor VIII **(A)**, bortezomib **(B)**, crizotinib **(C)**, phenformin **(D)**, vinorelbine **(E)** and pyrimethamine **(F)**. Correlation analysis between risk score and the IC50 of AKT inhibitor VIII **(G)**, bortezomib **(H)**, crizotinib **(I)**, phenformin **(J)**, vinorelbine **(K)** and pyrimethamine **(L)**.

In order to investigate the potential application of cuproptosis-related lncRNA prognostic signature in personalized therapy for DLBCL, we examined the association between the risk score and drug responsiveness. We evaluated the therapeutic response in each patient according to the half-maximal inhibitory concentration (IC50) of different drugs. Drug sensitivity analysis revealed that AKT inhibitor VIII, bortezomib, crizotinib, phenformin, vinorelbine and pyrimethamine showed significant sensitivity in the high- and low-risk groups. Patients in the low-risk group were more sensitive to AKT inhibitor VIII, bortezomib, crizotinib and phenformin than these in the high-risk group (Fig. 8A–D). And patients in the high-risk group were more sensitive to vinorelbine and pyrimethamine than these in the low-risk group (Fig. 8E, F). The correlation of risk score and drug sensitivity indicated that the risk score was significantly positively correlated with AKT inhibitor VIII (R = 0.23, *P* = 4.1e − 07), bortezomib (R = 0.16, *P* = 0.00051), crizotinib (R = 0.27, *P* < 8.4e − 09), phenformin (R = 0.25, *P* < 4.9e − 08), but negatively correlated with vinorelbine (R =  − 0.25, *P* < 5e − 08) and pyrimethamine (R =  − 0.21, *P* < 3.9e − 06) (Fig. 8G–L). The findings indicated that these antineoplastic drugs may play a promising role in the future treatment of DLBCL.

### Construction of PPI based on WGCNA

**Figure 9:**
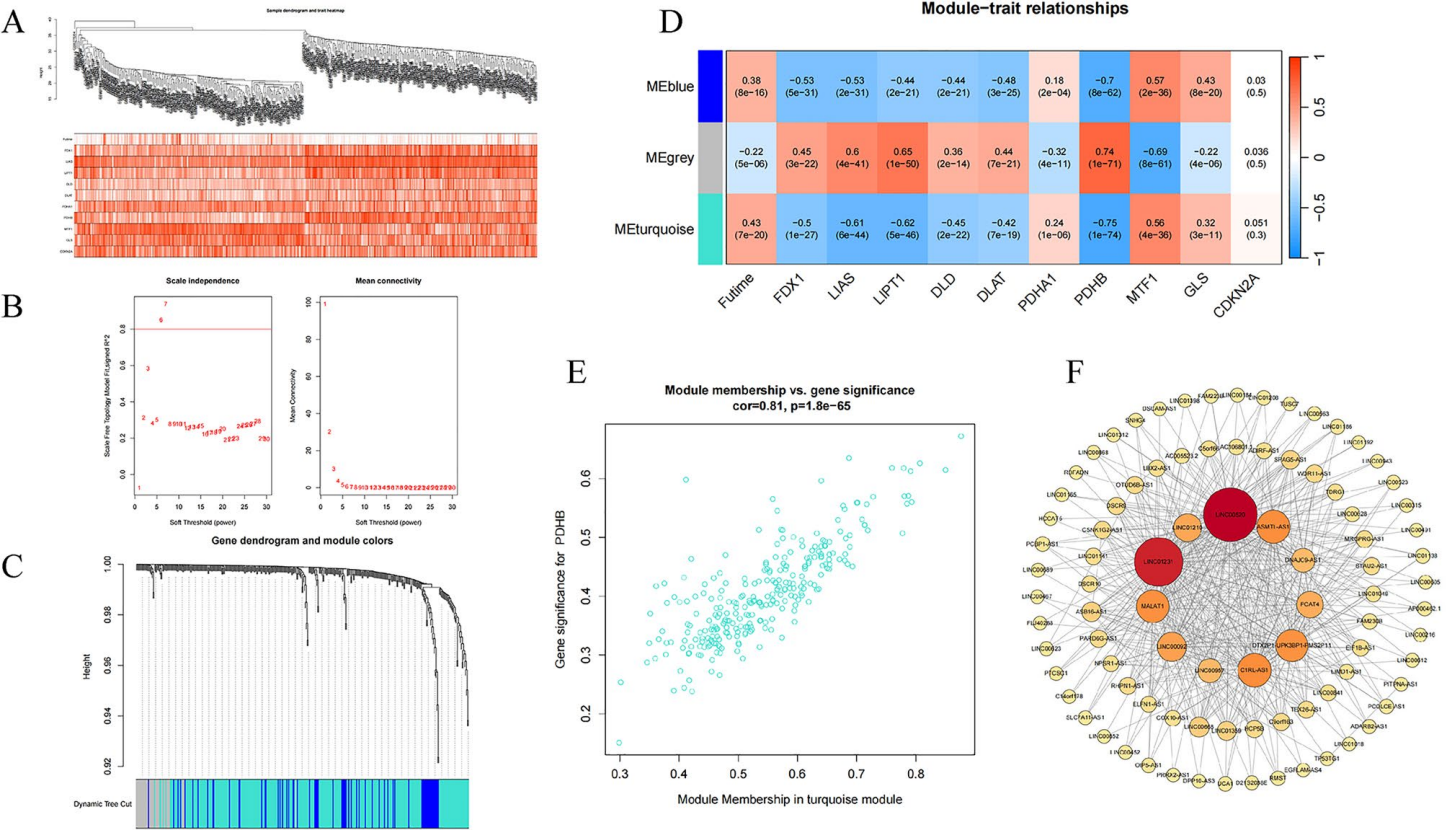
The WGCNA analysis of GSE10846 and identification of candidate hub genes. **(A)** The construction of cluster samples and trait information. **(B)** The soft threshold power and the mean connectivity of WGCNA. **(C)** The cluster dendrogram of WGCNA. **(D)** The clustered modules of WGCNA. **(E)** The scatter plots of the gene significance and module membership. **(F)** PPI network of 11 genes were obtained by Cytoscape.

WGCNA could effectively identify gene modules with similar expression patterns and hub genes with high connectivity. The cuproptosis-related lncRNA data were imported into R along with cuproptosis-related mRNA data and futime data for WGCNA analysis, enabling the construction of cluster samples and trait information (Fig. 9A), and then the relationship matrix and adjacence matrix were constructed in turn. The topological overlap matrix was constructed using a power value of 7 (Fig. 9B), yielding three co-expression modules (Fig. 9C). Subsequently, the correlation between these modules and traits was analyzed based on the Pearson correlation coefficient (Fig. 9D). The results of the module-trait relationship analysis reveal that the turquoise module exhibits a significantly negative correlation with PDHB (coefficient =  − 0.75, *P* = 1e − 74). The genes in the ME turquoise module were included for further analysis, and the gene significance and module membership were shown in scatter plots (Fig. 9E). The edges with the top 500 weight values were selected and imported into Cytoscape for the construction of a visualized PPI network. The results revealed that among the central genes in the network, LINC00520 and others ranked as the top eleven genes based on their degree values (Fig. 9F).

## Discussion

Due to the high heterogeneity of DLBCL in both biological and clinical features, there is significant variation in the prognosis of patients with DLBCL. At present, the IPI is the predominant method utilized to assess prognosis and guide treatment decisions for patients with DLBCL. Age and stage are the components of the IPI. Older age and later stage are associated with a larger IPI score and poorer prognosis. However, IPI relies only on clinical factors and may not provide a comprehensive forecast of DLBCL patient outcomes. The prognosis of DLBCL is affected by multiple factors including clinical characteristics, molecular subtype and the tumor microenvironment. No single biomarker can accurately identify the prognosis of DLBCL patients. Our study suggests that age, stage and gender do not impact the prognosis of DLBCL independently. Research shown that the effect of age on prognosis varies not only by NHL subtype, but also by gender within certain subtypes^26^. In treated DLBCL patients, efficacy and PFS were not affected by age^27^. The heterogeneity of NHL, the refinement of histopathological categorization, and the high incidence of advanced stage of lymphoma make the Ann Arbor classification of NHL insufficiently^28^. Only the advanced Ann Arbor stage and age over 60 years are two major factors predicting poor prognosis in patients with DLBCL subtype, but not as an independent prognostic factors of the OS in DLBCL patients^29^. IPI relies only on clinical factors and fails to fully capture the molecular biological heterogeneity involved in the occurrence and progression of DLBCL. Consequently, it cannot accurately stratify the risk and predict the prognosis of DLBCL. Therefore, it is urgent to develop novel prognostic evaluation models. Previous research has demonstrated that copper plays an anti-tumor role by regulating cell death^16^. Copper have been confirmed involved in the occurrence and development of malignant tumors, but few studies focused on cuproptosis-related lncRNAs. Therefore, our study aims to elucidate the role of cuproptosis-related lncRNAs in DLBCL progression, and develop a prognostic model which can indicate the prognosis of DLBCL patients more accurately.

Homologous lncRNAs can play different roles in different tumors. For example, LncRNA NEAT1 regulates pyroptosis in glioma cells and colorectal cancer cells by targeting miR-296-5p or miR-448; while its high expression promotes ferroptosis in non-small cell lung cancer^30^. Studies on the relationship between lncRNA and cuproptosis have been reported in various diseases. LncRNA MIR31HG can inhibit cuproptosis and promote the proliferation, migration, and invasion of lung adenocarcinoma cells by down-regulating miR-193a-3p and increasing downstream TNFRSF21 expression^31^. LncRNA XIST can sponge miR-92b-3p and regulate the cuproptosis-related gene MTF1 to influence the progression of breast cancer^32^. LncRNAs are valuable prognostic indicators for patients with tumor, and play important roles in initiation, development, and progression of DLBCL^33–35^. Our research suggests that a prognostic risk model based on cuproptosis-related lncRNAs can effectively predict the prognosis of DLBCL patients, offering practical implications for clinical use. Among the seven lncRNAs included in the risk model, LINC00654 has been identified as the hub lncRNA in DLBCL patients, and LncRNA00654-NINL mRNA regulatory axis could be involved in DLBCL progression^36^. The rapid onset of radiation-induced lymphoma observed in NINL transgenic mice confirms an oncogenic role for NINL gene^37^. Our data indicates that LINC00654 is a high-risk lncRNA in DLBCL, and decreasing its expression may inhibitor tumor growth. We hypothesize that increased levels of LINC00654 in DLBCL result in elevated expression of the NINL gene, which negatively regulates cuproptosis through modulation of cell cycle changes, thus promoting the progression of DLBCL. LINC00294 has been discovered as an oncogene in various tumors. For example, LINC00294 inhibits mitochondrial function and promotes glioma cell apoptosis via miR-21-5p/CASKIN1/cAMP axis^38^. Additionally, LINC00294 can promote cervical cancer development by promoting cell cycle transition^39^. Further investigation is needed to understand the involvement of LINC00294 in regulating cuproptosis. Significantly, we observed that LINC00294 expression was lower in the high-risk group than that in the low-risk group. WWC2-AS2, LINC00661 and RNF139-AS1 were identified as prognostic indicators in lung adenocarcinoma, cholangiocarcinoma and bladder cancer, respectively^40–42^. In our study, novel lncRNAs including LINC00294, WWC2-AS2, LINC00661, RNF139-AS1, LINC01165 and LINC01398 were identified in DLBCL for the first time. However, the regulatory connection between lncRNAs and cuproptosis has primarily been examined through database analysis, with limited experimental investigations conducted on these associations. The expression pattern, clinical relevance and underlying mechanisms of these lncRNAs in DLBCL patients is still poorly understood. Therefore, more studies are needed to elucidate the function of these lncRNAs in the pathogenesis of DLBCL.

The tumor microenvironment consists of tumor cells, infiltrating immune cells, bone marrow-derived inflammatory cells, fibroblasts, signaling molecules, peripheral blood vessels and extracellular matrix. Immune cell infiltration is a immunological feature of the tumor microenvironment and is essential for the development of inflammatory environment and immune escape of tumor cells. Previous studies on lymphoma suggested that all TME cells exhibit varying survival associations, which demonstrated context-dependent heterogeneity in clinical outcomes^43^. In this study, a notable increase in the proportion of immune cells, including regulatory T (Treg) cells, neutrophils, mast cells, eosinophils, γδ T cells, and type 2 helper T cells was observed in high-risk DLBCL group. The imbalance between immune effector cells and immune suppressive cells may lead to a disturbed immune microenvironment, leaving an immunosuppressed state in DLBCL. Treg cells has been identified as major contributors to a suppressive immune microenvironment, and the increased proportion of Treg cells are associated with worse outcomes in DLBCL^44,45^. The proportion of Treg cells in peripheral blood may be good prognostic markers for OS after 2 years in relapsed refractory DLBCL patients^46^. The neutrophil/lymphocyte (N/L) ratio at diagnosis has been proved to be a prognostic factor for survival in DLBCL, and patients with an N/L ratio < 3.5 at diagnosis experienced a superior OS compared with those with an N/L ratio ≥ 3.5 at diagnosis^47^. Higher neutrophils and N/L ratio may indicate a poorer prognosis for DLBCL patients at diagnosis^48^. These findings are highly consistent with our results. In summary, the cuproptosis-related lncRNA-based risk model may play an important role in indicating immune cell infiltration of DLBCL. The immune regulatory mechanisms by which the above cells infiltration contributes to tumor invasion and progression in DLBCL need to be further investigated.

Through functional enrichment analysis, we found that differentially expressed genes between the low-risk and high-risk groups were mainly enriched in cell metabolic pathways, including phospholipid metabolic pathways, cellular carbohydrate metabolic pathways, regulation of carbohydrate biosynthetic process, and primary bile acid biosynthesis pathways. Mitochondrial metabolism plays a central role in cell metabolism, and many metabolic pathways such as the tricarboxylic acid cycle (TCA), oxidative phosphorylation, fatty acid oxidation, nucleotide synthesis and amino acid metabolism occur in mitochondria. Research shows that disruptions in copper homeostasis can lead to aberrant metabolic process^49,50^.Mitochondrial respiration has been considered as vital process in cuproptosis^16^. This suggests that there may be differences in mitochondrial metabolism between the high-risk and low-risk groups, which may be one of the potential reasons for the different prognoses between the two groups. Furthermore, differentially expressed genes were also enriched in signal transduction, inflammation pathways, such as response to ROS, MAPK signaling pathway, HIF-1 signaling pathway, Ras signaling pathway. These pathways have been found to be involved in the progression and therapeutic response of DLBCL. For example, TEOA inhibits tumor proliferation and induces DNA damage of DLBCL cells through activation of the ROS-dependent p38 MAPK signaling pathway^51^. The activation of HIF-1α results in suppression of translation under hypoxic stress in DLBCL^52^. HIF-1 expression predicts superior survival in patients with DLBCL treated with R-CHOP^53^. Apatinib exerts anti-tumor activity by inhibition of the Ras pathway in DLBCL^54^. These may be potential targets for prevention and treatment of DLBCL. The underlying mechanism how cuproptosis-related lncRNA induces cuproptosis in DLBCL by the above signaling pathways still needs further investigation.

We found that the IC50 value of most drugs increased with the increase of risk score, which suggested that patients in high-risk group may develop resistance to most anticancer drugs. Our study also indicated that high-risk patients may be sensitive to vinorelbine and pyrimethamine. R-CHOP chemotherapy is the standard treatment for patients with DLBCL, but vincristine induced peripheral neuropathy, which occurs in more than a third of the patients, might require earlier treatment discontinuation^55^. Vinorelbine induces cell cycle arrest in the G2-M phase, its inhibition leads to premature mitotic entry and cell death. Studies have shown that replacement of vincristine by vinorelbine due to neuropathy is effective and safe, which significantly improve neuropathy in patients compared to treatment with R-CHOP^56^. The regimen including rituximab, vinorelbine, ifosfamide, mitoxantrone and prednisone displays significant activity and acceptable toxicity in relapsed DLBCL^57^. The combination of prednisolone, vinorelbine, etoposide, cyclophosphamide and rituximab dramatically increases the 24-months disease free survival (74%) in vulnerable elderly NHL patients as compared to treatment with mini R-CHOP (60%)^58^. So far, the application of pyrimidine in DLBCL has not been reported. In conclusion, vinorelbine and pyrimidine may be potential therapeutic targets for DLBCL, which provides the basis for precise individualized treatment for DLBCL patients.

However, there are still limitations in this study. Firstly, the number of DLBCL samples in TCGA and GSE10846 data is not enough, and we need additional data from different databases for external validation of our model. Secondly, the cuproptosis-related lncRNAs that can guide DLBCL prognosis and the immune microenvironment obtained in this study require further research into the biological function.

## Conclusions

In summary, we have developed a risk model incorporating a signature of cuproptosis-related lncRNA that demonstrates independent prognostic value for DLBCL.In addition, our preliminary analysis has indicated a relationship between the risk model and immunological characteristics, which offers insights into the underlying mechanism of cuproptosis and suggests individual and precise therapeutic strategies in DLBCL. The cuproptosis-related lncRNA-based risk model integrates the clinical characteristics, molecular biomarkers, immune microenvironment on the prognosis of DLBCL patients, which provides a new perspective for the stratified diagnosis of DLBCL. However, this model does not explicitly consider the effect of gene mutation factors on the prognosis of patients. In addition, it is important to acknowledge the limitations of this model, such as sample size, potential confounders, and the external validity. Future studies will therefore be necessary to validate our findings in independent cohorts and through experimental validation.

### Supplementary Information

Supplementary Figure 1.

## Data Availability

The datasets generated and analyzed during the current study are not publicly available due to correlation with some unpublished researches, but are available from the corresponding author on reasonable request.
