# Supplementary figures and images for: Cuproptosis-related lncRNA signature as a prognostic tool and therapeutic target in diffuse large B cell lymphoma
Source: Sci Rep. 2024 Jun 5;14:12926. doi: 10.1038/s41598-024-63433-w (PMC11153514; doi:10.1038/s41598-024-63433-w)

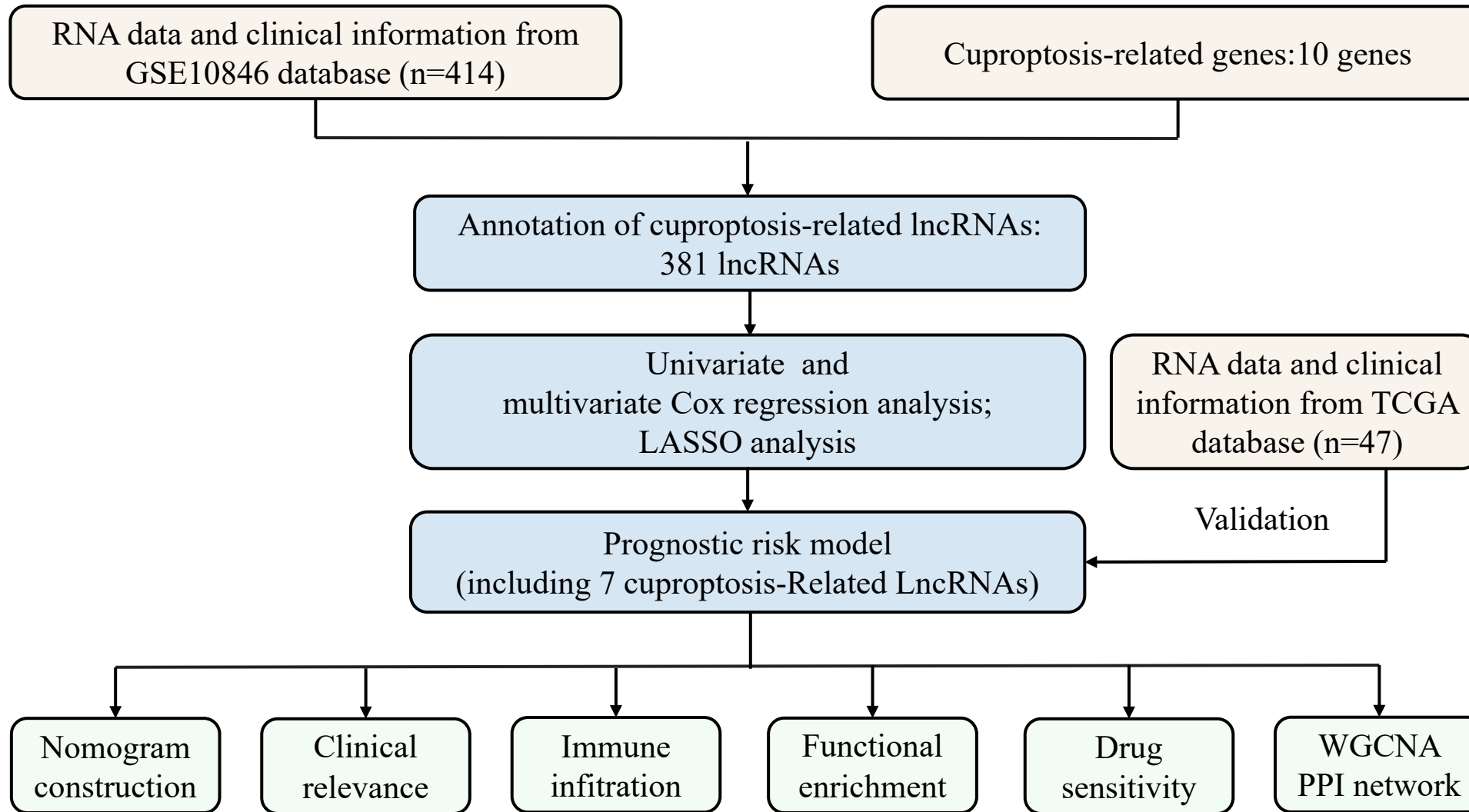

**Supplementary Figure 1** The flow chart diagram of the study.

Supplement: Supplementary file 1 — Supplementary Figure 1. [file 41598_2024_63433_MOESM1_ESM.pdf]
